# Extra-adrenal pheochromocytoma with initial symptom of haemoptysis: a case report and review of literature

**DOI:** 10.1186/s12893-020-01038-6

**Published:** 2021-01-06

**Authors:** Yutaka Endo, Minoru Kitago, Masahiro Shinoda, Hiroshi Yagi, Yuta Abe, Shutaro Hori, Masanori Odaira, Takahiro Yokose, Kaori Kameyama, Yuko Kitagawa

**Affiliations:** 1 Department of Surgery, Keio University School of Medicine, Tokyo, 1608582 Japan; 2 Department of Surgery, Tokyo Saiseikai Central Hospital, Tokyo, Japan; 3 Department of Pathology, Keio University School of Medicine, Tokyo, Japan

**Keywords:** Extra-adrenal abdominal pheochromocytoma, Haemoptysis, Paraganglioma

## Abstract

**Background:**

Pheochromocytoma is a catecholamine-secreting tumour that leads to various symptoms. Haemoptysis is rarely caused by a pheochromocytoma occurring outside the bronchus or thoracic cavity. Here, we report the case of an extra-adrenal abdominal pheochromocytoma initially manifesting as haemoptysis/dyspnoea during exercise without classic symptoms.

**Case presentation:**

A 22-year-old man with a history of severe dyspnoea experienced difficulties in breathing following a marathon owing to haemoptysis that required ventilator management 1 year before presentation. His father had undergone surgery for ectopic pheochromocytoma. Computed tomography (CT) revealed a 30-mm tumour between the inferior vena cava and pancreatic head while urinalysis revealed abnormally high noradrenaline levels. He was clinically diagnosed with an extra-adrenal abdominal ectopic pheochromocytoma. After controlling blood pressure, surgery was performed, and the tumour was successfully removed. Histopathology revealed chromogranin A (+), synaptophysin (+), S100 protein (+), and MIB-1 index of 1%. Therefore, the patient was finally diagnosed with extra-adrenal abdominal ectopic pheochromocytoma.

**Conclusions:**

Haemoptysis is a rare manifestation of abdominal ectopic paraganglioma. Prompt consideration of pheochromocytoma/paraganglioma when patients experience haemoptysis without any other possible aetiology may prevent inappropriate diagnosis and treatment and ultimately fatalities.

## Background

Catecholamine-secreting tumours derived from chromaffin cells of the adrenal medulla and sympathetic ganglia are referred to as pheochromocytomas and extra-adrenal pheochromocytomas, respectively. Pheochromocytoma/ectopic pheochromocytoma (hereinafter, “paraganglioma”) exhibits various symptoms based on abnormal hormone production [1]. However, unless it occurs in the bronchus or thoracic cavity, it rarely causes hemoptysis [2]. Herein, we describe a case of intraabdominal paraganglioma with haemoptysis as an initial symptom.

## Case presentation

A 22-year-old man presented with a history of breathing difficulty during exercise along with haemoptysis. One year prior, he had experienced a similar episode of breathing difficulty, requiring ventilator management owing to haemoptysis. Computed tomography (CT) at that time indicated diffuse alveolar haemorrhage, and echocardiography showed severe apical asynergy. He was successfully treated with supportive care. His family history was significant with his father’s undergoing ectopic paraganglioma resection.

**Fig. 1:**
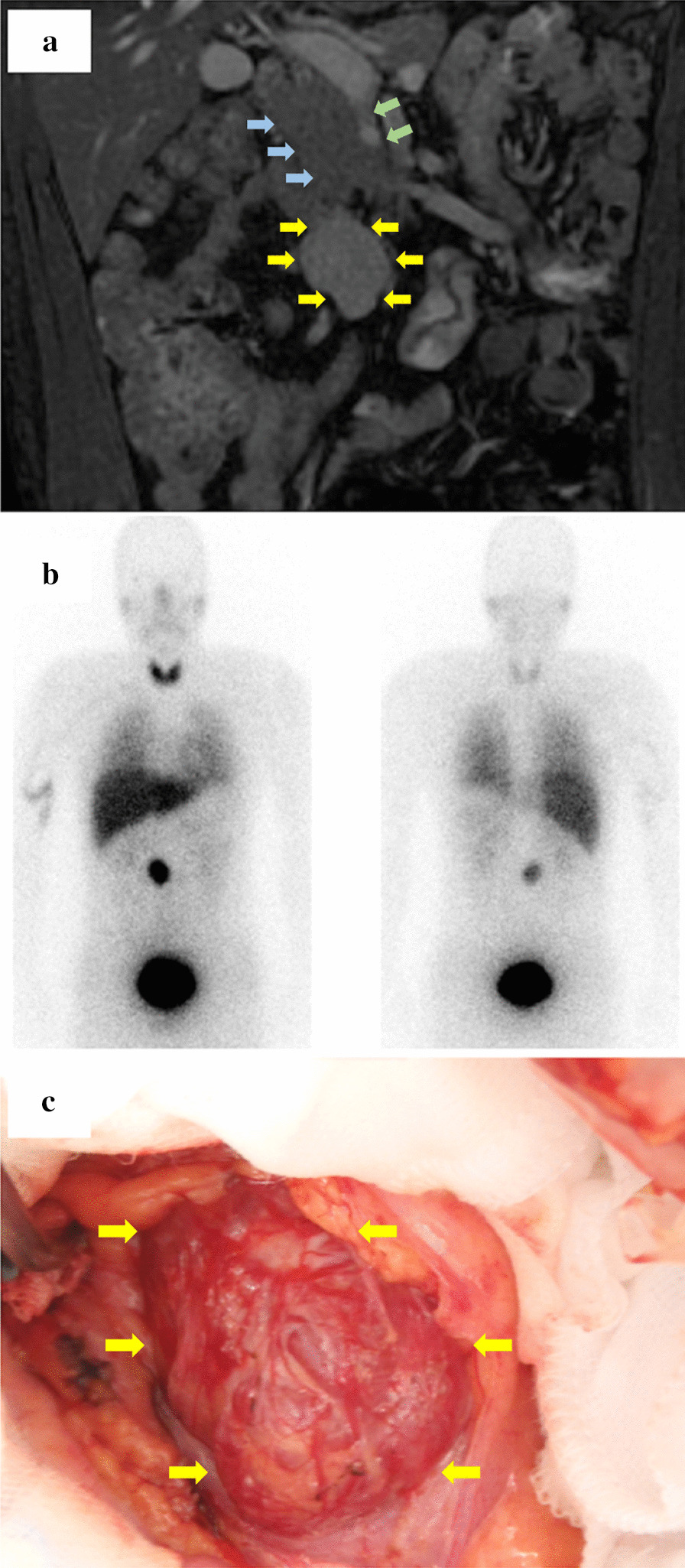
Radiological and intraoperative findings of the lesion. T2-weighted coronal MRI of the abdomen shows a heterogeneous mass (yellow arrows) that is slightly hyperintense to muscle between the inferior vena cava (IVC, blue arrows) and the head of the pancreas. The supra mesenteric vein (SMV, green arrows) runs ventrally near the tumour (**a**). MIBG scintigraphy reveals a strong accumulation at the same location detected on MRI (**b**). During surgery, a 30-mm tumour (yellow arrows) is found below the retroperitoneum on the caudal side of the mesocolon (**c**). MRI, magnetic resonance imaging; MIBG, metaiodobenzylguanidine,

His blood pressure was normal without other specific symptoms. Past medical history was indicative of cardiomyopathy induced by excessive catecholamine. Further investigation revealed an abnormally high serum noradrenalin value (1.70 ng/mL) and normetanephrine in the urine (1.6 mg/day upon 24-h collection). Other laboratory data, including plasma catecholamine and urinary vanillylmandelic acid levels, were normal. Radiological examination did not reveal any respiratory lesions; however, contrast CT unexpectedly indicated lobular tumorous lesions with internal homogeneity that enhanced aridly, followed by a delayed anterior inferior vena cava (IVC) washout. T2-weighted magnetic resonance imaging (MRI) revealed a 28 × 32 × 50 mm tumour with slightly higher intensity than the muscle between the IVC and pancreatic head (Fig. 1a). Metaiodobenzylguanidine (MIBG) scintigraphy revealed strong accumulation at the site, which was consistent with presence of a tumour (Fig. 1b), leading to a diagnosis of paraganglioma. After blood pressure stabilization (within 130/80 mmHg) with alpha-adrenergic antagonist and doxazocin, tumour resection was performed. An appropriate volume of fluids was administered in the hour preceding the resection. During surgery, a 30-mm tumour was found below the retroperitoneum on the caudal side of the mesocolon (Fig. 1c). Despite intraoperative blood pressure fluctuations of up to 200 mmHg, surgery was completed without significant complications. Anaesthesia time, operation time, blood loss, and urine output during the surgery were 210 min, 110 min, 10 mL, and 1250 mL, respectively.

**Fig. 2:**
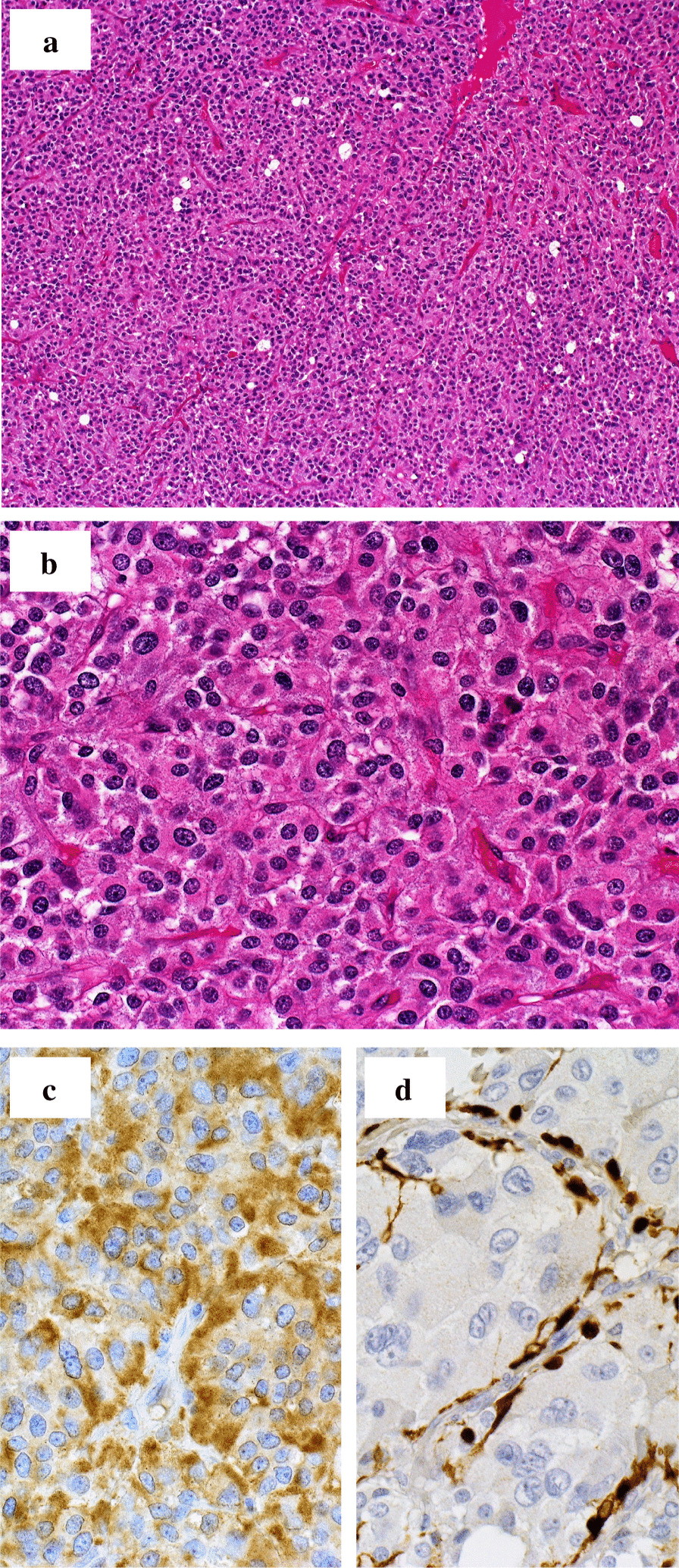
Pathological findings of the resected specimen. Haematoxylin and eosin (HE) staining lower-power field (**a**) and high-power field (**b**). Immunohistochemical staining of chromogranin A (**c**) and S100 protein (**d**) reveals strong reactivity in the sustentacular cell

His postoperative recovery was generally good, and he was discharged from the hospital on postoperative day 8. Pathological findings demonstrated a well-circumscribed, lobulated mass 42 × 28 mm in size. The microscopic findings showed diffuse patterns of clustered oval cells abundant with eosinophilic cytoplasm separated by delicate fibrovascular stroma. Immunostaining was synaptophysin-positive, S100 protein-positive in the supporting cells, and chromogranin A-positive, leading to a diagnosis of paraganglioma (Fig. 2a–c). Hormone levels returned to normal after surgery. No malignant findings or any apparent signs of recurrence were observed during the 5-year follow-up period since surgery.

## Discussion and conclusions

We describe an unusual clinical course of ectopic paraganglioma in a patient who previously developed acute respiratory failure owing to massive haemoptysis.

Because the clinical signs and symptoms of paraganglioma are commonly described together with those of pheochromocytoma, the exact incidence of paraganglioma has not been investigated. In 2009, approximately 3000 pheochromocytomas and paragangliomas were diagnosed in Japan [3]. Most paragangliomas are benign, and malignant paragangliomas are rare. The mean age at diagnosis was 47 years in a study on benign paragangliomas [4]. Patients with genetic predisposition are inclined to develop the disease about a decade earlier than those without. In about one-third of cases [5], a paraganglioma is one component of an inherited syndrome [6]. Most cases of hereditary paraganglioma are associated with mutations in the genes encoding different subunits of the succinate dehydrogenase enzyme complex, *RET*, *NF1,* and *VHL*. Given that this patient was young at presentation with a paternal genetic predisposition, genetic testing was considered; nonetheless, he declined to undergo testing.

Generally, paragangliomas are asymptomatic at presentation. With more widespread use of cross-sectional imaging, asymptomatic pheochromocytomas and paragangliomas may be discovered incidentally on imaging studies performed for other reasons. When symptomatic, clinical presentation depends on tumour location, catecholamine secretion, and other factors. Extra-adrenal paragangliomas can arise within various sites in the skull base and neck, thorax, or abdomen and pelvis, exhibiting a mass effect. Paragangliomas outside of the skull base and neck rarely present with mass effect symptoms. As in pheochromocytomas, paragangliomas lead to symptoms of catecholamine excess. Hypertension (often paroxysmal) is frequently associated with episodic headache, sweating, and palpitations, referred to as the “classic triad” [1]. Our patient exhibited neither the classic triad nor other symptoms associated with excessive catecholamine secretion and mass effect. Rarely, pheochromocytoma is associated with cardiomyopathy attributed to catecholamine excess comparable to stress-induced (takotsubo) cardiomyopathy. The imbalance between myocardial oxygen supply and demand is believed to be the key to the pathophysiology [7]. There have been five previous reports of pheochromocytoma/paraganglioma patients presenting with hemoptysis [2, 8–11]. According to these reports, pulmonary venous hypertension owing to paroxysmal hypertension may result in hemoptysis [9]. Furthermore, it has been reported that catecholamine production may cause endothelial dysfunction and abnormal clotting [7].

Endocrinological laboratory testing, including urinary and plasma-fractionated metanephrine and catecholamine levels, is indicated for all paragangliomas, regardless of whether it is clinically manifested or not. Radiologic imaging, including CT, MRI, and MIBG scintigraphy, are essential assessments. Octreoscans are also a useful modality for detecting paragangliomas. If metastatic diseases are suspected, 18-fluorodeoxyglucose or Ga-68-Dotatate positron emission tomography/CT are recommended. Based on imaging findings, the physicians can decide whether the tumour is resectable or metastatic. Complete *en bloc* surgical resection may cure all potentially resectable paragangliomas. Resecting a catecholamine-secreting tumour is a high-risk procedure, and an experienced surgeon/anaesthesiologist team is required. Before surgery, it is essential for the surgeon to assess the adequacy of the adrenergic blockade. During the procedure, the surgeon should frequently communicate with the anaesthesiologist, especially upon incision, at the time of division of the venous supply, and during tumour manipulation. During surgery, cardiovascular and hemodynamic variables must be monitored closely, with continuous measurement of intra-arterial pressure and heart rhythm. Laparoscopic resection is the preferred surgical approach of experienced surgeons; however, we chose an open procedure for this patient because a laparoscopic approach is contraindicated for patients with large tumours [12]. Some patients with an inherited syndrome predisposing them to paraganglioma may have multiple concurrent tumours (e.g., von Hippel-Lindau disease). In general, treatment of a catecholamine-secreting tumour has priority and is resected first to achieve hemodynamic stability. Because simultaneous resection of catecholamine-secreting tumours and other concurrent neoplasms often leads to a high complication rate, two-stage resection might be appropriate for multiple tumours [13]. There are no large studies on specific outcomes of paragangliomas located in the abdominal cavity. In one study of patients with pheochromocytoma or secretory extra-adrenal paraganglioma, the 5-year likelihood of recurrence among those with an extra-adrenal paraganglioma was approximately 20% [14]. Our patient had no signs of recurrence during the 5-year follow-up period after surgery.

In conclusion, our experience with this patient was unique because he did not exhibit typical symptoms, such as high blood pressure or headaches, making initial diagnosis difficult. As massive haemoptysis and paraganglioma are medical emergencies, it is important to consider pheochromocytoma/paraganglioma when patients experience haemoptysis that cannot be associated with other possible aetiologies.

## Data Availability

This is not applicable to this article because this is a case report.

## Abbreviations

CT: Computed tomography
IVC: Inferior vena cava
MRI: Magnetic resonance imaging
MIBG: Metaiodobenzylguanidine
